# Changes in alpha, theta, and gamma oscillations in distinct cortical areas are associated with altered acute pain responses in chronic low back pain patients

**DOI:** 10.3389/fnins.2023.1278183

**Published:** 2023-10-13

**Authors:** George Kenefati, Mika M. Rockholt, Deborah Ok, Michael McCartin, Qiaosheng Zhang, Guanghao Sun, Julia Maslinski, Aaron Wang, Baldwin Chen, Erich P. Voigt, Zhe Sage Chen, Jing Wang, Lisa V. Doan

**Affiliations:** ^1^Department of Anesthesiology, Perioperative Care and Pain Management, New York University Grossman School of Medicine, New York, NY, United States; ^2^Interdisciplinary Pain Research Program, New York University Grossman School of Medicine, New York, NY, United States; ^3^Department of Otolaryngology-Head and Neck Surgery, New York University Grossman School of Medicine, New York, NY, United States; ^4^Department of Psychiatry, New York University Grossman School of Medicine, New York, NY, United States; ^5^Department of Neuroscience and Physiology, Neuroscience Institute, New York University Grossman School of Medicine, New York, NY, United States; ^6^Department of Biomedical Engineering, New York University Tandon School of Engineering, Brooklyn, NY, United States

**Keywords:** chronic pain, electroencephalography, source localization, oscillations, alpha, theta, gamma

## Abstract

**Introduction:**

Chronic pain negatively impacts a range of sensory and affective behaviors. Previous studies have shown that the presence of chronic pain not only causes hypersensitivity at the site of injury but may also be associated with pain-aversive experiences at anatomically unrelated sites. While animal studies have indicated that the cingulate and prefrontal cortices are involved in this generalized hyperalgesia, the mechanisms distinguishing increased sensitivity at the site of injury from a generalized site-nonspecific enhancement in the aversive response to nociceptive inputs are not well known.

**Methods:**

We compared measured pain responses to peripheral mechanical stimuli applied to a site of chronic pain and at a pain-free site in participants suffering from chronic lower back pain (*n* = 15) versus pain-free control participants (*n* = 15) by analyzing behavioral and electroencephalographic (EEG) data.

**Results:**

As expected, participants with chronic pain endorsed enhanced pain with mechanical stimuli in both back and hand. We further analyzed electroencephalographic (EEG) recordings during these evoked pain episodes. Brain oscillations in theta and alpha bands in the medial orbitofrontal cortex (mOFC) were associated with localized hypersensitivity, while increased gamma oscillations in the anterior cingulate cortex (ACC) and increased theta oscillations in the dorsolateral prefrontal cortex (dlPFC) were associated with generalized hyperalgesia.

**Discussion:**

These findings indicate that chronic pain may disrupt multiple cortical circuits to impact nociceptive processing.

## 1. Introduction

Pain is a dynamic and multi-dimensional experience shaped by sensory, affective, and cognitive components (Melzack, 2001). Chronic pain, meanwhile, is characterized by both an altered brain state and an abnormal response to transient, evoked inputs (Apkarian et al., 2005; Tracey and Bushnell, 2009; Apkarian et al., 2011). Chronic pain has well-known associations with symptoms of allodynia and/or hypersensitivity at the site of tissue or nerve injury (Basbaum et al., 2009; Latremoliere and Woolf, 2009). At the same time, studies from pain syndromes such as fibromyalgia and knee osteoarthritis have shown that the presence of chronic pain also causes increased pain severity and a distorted pain intensity scale in a diffuse non-anatomic pattern (Petzke et al., 2003; Kudel et al., 2007; Rakel et al., 2015). Thus, chronic pain not only causes hypersensitivity at sites of tissue injury, but it can also lead to a generalized form of enhancement in aversion to evoked stimuli.

Previous neuroimaging studies based on functional magnetic resonance imaging (fMRI) and positron emission tomography (PET) have revealed that chronic pain causes maladaptive changes in a distributed cortical network including the primary somatosensory cortex (S1), the anterior cingulate cortex (ACC), dorsolateral prefrontal cortex (dlPFC), and the insular cortex (IC; Apkarian et al., 2004, 2005; Brooks and Tracey, 2005; Tracey, 2005; Tracey and Mantyh, 2007; Kucyi and Davis, 2015; Mouraux and Iannetti, 2018; Prichep et al., 2018; Van Der Miesen et al., 2019; Chen, 2021). These findings are further supported by studies in animal models (Bingel and Tracey, 2008; Zhang et al., 2017; Xiao et al., 2019; Sun et al., 2023). Less is known, however, about how the brain, in particular the cortex, dynamically responds to a temporally regulated stimulus (Wager et al., 2013; Sun et al., 2021). Electroencephalography (EEG) provides recordings of oscillatory activity in the cortex both at rest and in response to noxious input with high temporal resolution (Hutchison et al., 1999; Pinheiro et al., 2016; Ploner and May, 2018; Levitt and Saab, 2019; Davis et al., 2020). Resting-state EEG studies have shown both increased and decreased power in peak alpha frequency and theta bands, increased beta-band power with increased event-related desynchronization (ERD) in the same bands, mainly in the frontal, parietal, and occipital cortices, and these studies are in general agreement with fMRI and PET results (De Vries et al., 2013; González-Roldán et al., 2016; Levitt et al., 2020; Rockholt et al., 2023). Meanwhile, recent studies of evoked EEG potentials in response to acute painful stimuli, especially in the context of pre-existing chronic pain (Teixeira M. et al., 2021; Teixeira P. E. P. et al., 2021), allow for enhanced understanding of the mechanisms underlying nociception and hyperalgesia (Plaghki and Mouraux, 2005). These studies have shown that chronic pain causes neurons to undergo long-term plasticity that can manifest as increased spiking rates or increased power in high gamma (>60 Hz) and theta (4–8 Hz) oscillations in response to noxious stimuli (Babiloni et al., 2002; Wang et al., 2011; Schulz et al., 2012a; Misra et al., 2017; Zhou R. et al., 2018; Dinh et al., 2019; May et al., 2019; Vanneste et al., 2019; Vanneste and De Ridder, 2021). Some of these studies have applied source localization to infer the cortical sources for such oscillatory changes, showing that both chronic pain and acute pain are associated with abnormalities in regions such as the ACC (Babiloni et al., 2002; Vanneste et al., 2019; Vanneste and De Ridder, 2021). However, few studies have carefully examined source-localized oscillatory changes in response to stimuli targeting painful and non-painful sites in chronic pain conditions. Such studies, however, are critical for understanding brain mechanisms for impairments of endogenous pain processing leading to abnormal pain behaviors.

We conducted a prospective study to identify behavioral and electrophysiological responses to acute mechanical stimuli applied to participants with chronic low back pain (CLBP) as well as pain-free control participants. Our results show that participants with chronic pain demonstrate more intense pain in both their back and the dorsum of the hand. Further, when we analyzed source localized EEG data, we found that hypersensitivity to back is associated with enhanced theta and alpha oscillations in the contralateral medial orbitofrontal cortex (mOFC), whereas increased response to peripheral mechanical stimulation to hand correlates with increased gamma oscillations in the contralateral ACC and increased theta oscillations in the contralateral dlPFC. These results suggest distinct mechanisms for site-specific hypersensitivity and more generalized hyperalgesia.

## 2. Methods

### 2.1. Ethical considerations and participants

The study was approved by the New York University Grossman School of Medicine Institutional Review Board (8/22/2019, #i19-01088) and conducted in accordance with the latest version of the Declaration of Helsinki. Written informed consent was obtained from all participants before study enrollment.

### 2.2. Eligibility criteria

A total of 15 participants with CLBP and 15 control participants were recruited in this study. For participants with CLBP, inclusion criteria were diagnosis of CLBP, defined as pain lasting greater than 6 months, with a baseline average back pain intensity >4 on a 0–10 numerical rating scale OR pain-free participants, aged between 18 and 75 years, and ASA physical status 1–3. Exclusion criteria included acute lumbosacral radiculopathy with signs such as sensory loss, motor weakness, and decreased reflexes, low back pain with any systemic signs or symptoms, cognitive impairment (by history) or clinical signs of altered mental status, history of schizophrenia, daily benzodiazepine use, and pregnancy.

### 2.3. Mechanical stimulation and pain assessment

Participants were blindfolded by a mask and asked to stay relaxed and in a wakeful state during the behavioral tasks and EEG recordings. Weighted mechanical pinprick stimulators (MRC Systems GmbH, Heidelberg, Germany) exerting forces of 32 mN and 256 mN were used. Pinpricks were applied both to the lower back (in the painful area in participants with CLBP) and the dorsum of the right hand. A total of ~10 trials per force were applied at each site, and stimulations were delivered in random order with an interstimulus interval of approximately 10 s. Participants were asked to rate each stimulus on a 0–10 numeric rating scale.

### 2.4. EEG recordings

Brain activity was recorded using a high density-EEG cap which includes four integrated bipolar leads for vertical and horizontal electrooculogram (EOG; 64-channel Quik-Cap Neo Net, Compumedics Neuroscan, Charlotte, NC, United States). The ground electrode was placed on the left cheek. The EEG cap was connected to the 64-channel Neuroscan SynAmps 2/RT and Nuevo Amplifier (Compumedics Neuroscan, Charlotte, NC, United States). For each session, two 5-min baseline recordings (one with eyes open and one with eyes closed) were performed before applying mechanical stimulations. All data was recorded using Curry 8 (Compumedics Neuroscan, Charlotte, NC, United States) at 1000 Hz.

### 2.5. EEG preprocessing

MNE-Python (version 1.0.3) was used for preprocessing (Gramfort et al., 2013; Larson et al., 2023). First, raw signals were down-sampled to a rate of 400 Hz and band-pass filtered between 1.0 and 100 Hz. A band-stop filter between 55 and 65 Hz removing line noise was applied. Further, noisy EEG channels were detected using PyPREP and subsequently interpolated in MNE-Python (Bigdely-Shamlo et al., 2015; Appelhoff et al., 2022). Criteria for noisy channel detection included low signal-to-noise ratio (SNR), lack of correlation with other channels, low or high relative deviations, presence of high-frequency noise, and poor prediction by other channels based on the random sample consensus approach. All signals were re-referenced to the average reference. An independent component analysis (ICA) based on the fast ICA algorithm was then conducted on the basis of the −0.2 to 0.8 s peri-stimulus time windows of the EEG data using a number of independent components (ICs) equal to half the number of EEG channels (Hyvarinen, 1999). Subsequently, ICs representing artifacts originating from eye movements recorded in the EOG electrode were removed from the EEG data. The cleaned data were analyzed using functions in MNE-Python as well as custom-written Python code. Data were segmented into epochs ranging from −0.2 to 0.8 s in peri-stimulus time. Noisy epochs were detected using the AutoReject package based on Bayesian optimization and were automatically marked for rejection (Jas et al., 2017). Following comparison to recording notes, automatically rejected epochs accurately matched trials marked as containing movement. For the 256 mN stimulation of the back, this resulted in a total of 144 trials for chronic pain and 143 trials for pain-free control participants. The numbers are 151 and 143 trials for the 32 mN stimulation for CLBP and pain-free participants, respectively. For the 256 mN stimulation of the hand, this resulted in 156 trials for chronic pain participants and 148 trials for control participants. The numbers are 156 and 147 trials for the 32 mN stimulation for CLBP and pain-free participants, respectively.

### 2.6. Source model

To project sensor-space time series to source space, we used dynamical statistical parametric mapping (dSPM) for noise-normalization, implemented in MNE-Python (Dale et al., 2000). The surface-based, three-shell boundary element model used for anatomical reconstruction is sourced from ‘fsaverage’, a template brain based on 40 MRI scans of real brains (Fischl et al., 1999, 2004; Destrieux et al., 2010). For each individual, a regularized noise covariance matrix was computed from a two-minute period in the resting condition before the start of the stimulus portion of the EEG recording. The inverse solution computed with a loose-orientation value of 0.2 was chosen to allow source space dipoles to have somewhat free orientation, but not too far from an orientation that is perpendicular to the cortex.

### 2.7. Assessment of source-space TFRs

Source-space time-frequency representations (TFRs) were obtained from the source time course epochs using a Slepian multitaper approach from 1 to 100 Hz, omitting the 55 to 65 Hz band-stop range. We selected event-related synchronization and desynchronization (ERS/ERD) as the preferred method for assessing changes in oscillatory activity following mechanical stimulation. ERS/ERD is defined as the percentage of power decrease or increase, respectively, according to the expression ERD% = (A − R)/R × 100 where the power within the frequency bands of interest in the period after the event is given by A and that that of the preceding baseline period is given by R (Pfurtscheller and Lopes Da Silva, 1999). Therefore, TFRs were referenced to the baseline period (−0.2 to 0.0 s) by subtracting the mean of the baseline and dividing by the mean of the baseline, resulting in percent-change power values. To compare responses to stimuli between the two groups, the source-space TFRs were first band-pass filtered to the frequency band of interest, theta (4 to 8 Hz), alpha (8 to 13 Hz), and high-gamma (65 to 100 Hz; May et al., 2019; Shirvalkar et al., 2023). We then averaged power values across time from 0.0 to 0.8 s post-stimulus onset for each trial of all participants in each group. To maximize the signal-to-noise ratio for visualization, TFRs represented by heat maps were averaged across epochs of the same stimulus site.

### 2.8. Statistical analyses

All data was analyzed using IBM SPSS Statistical software (Version 28, IBM, New York, United States) and GraphPad Prism (Version 9.4.1, GraphPad Software, Boston, United States). Results were expressed as mean ± standard error of mean (SEM) or median [interquartile range] for continuous variables and frequency and percentage for categorical variables. For all behavioral data, a non-parametric Mann–Whitney U test was used for hypothesis testing to compare the mean pain scores. A non-parametric Mann–Whitney U test was also used for hypothesis testing to compare the mean power values. We chose the Mann–Whitney U test to account for non-Gaussian distributions in the mean power values from the trials of each participant. The mean power in each frequency band was tested independently. *p* < 0.05 was considered significant.

## 3. Results

### 3.1. Participant characteristics

The sample (*n* = 30) consisted of age and gender-matched participants with CLBP [*n* = 15; 11 male (73%), 4 female (27%)], with the majority suffering from chronic pain for 1–5 years (60%), and those who were pain-free [*n* = 15: 11 male (73%), 4 female (27%)] with the mean age of 49.9 ± 4.71 and 50.5 ± 3.76, respectively.

### 3.2. Participants with CLBP experience increased sensitivity at painful and non-painful sites

**Figure 1:**
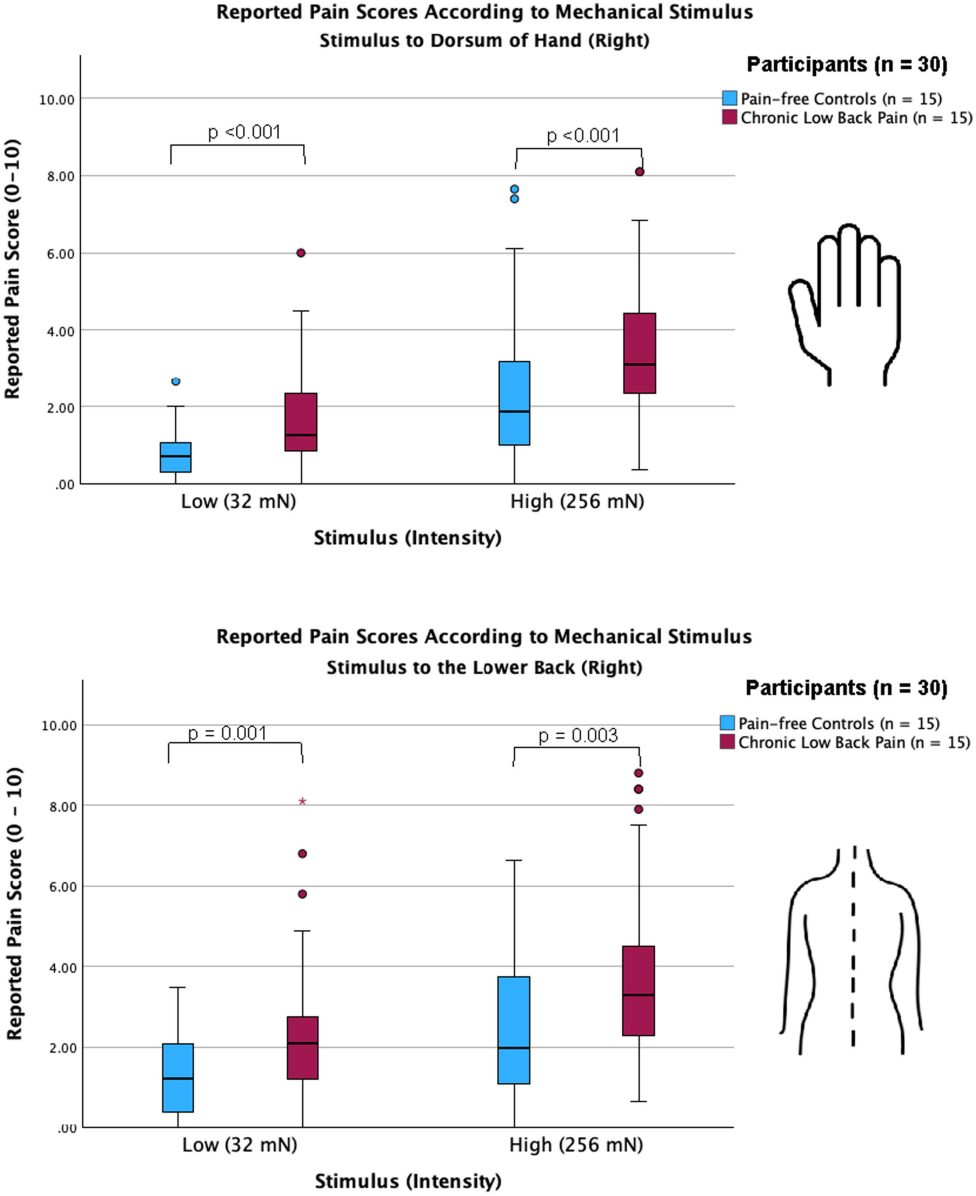
Chronic low back pain participants show increased sensitivity in both back and hand compared with pain-free controls. Clustered box plots with median [interquartile range] for pain scores (numerical rating scale, 0–10) for stimuli applied to the dorsum of right hand (upper graph) and the lower back (lower graph) of pain-free controls and chronic pain participants. Participants with chronic lower back pain, as well as pain-free control participants, reported significantly higher pain scores for both low-intensity (32 mN) and high-intensity stimuli (256 mN) when applied to the dorsum of the hand (*p* < 0.001) and the lower back (*p* = 0.001 and *p* = 0.003, respectively). Outliers are represented by red circles and stars. Hypothesis testing was performed using the non-parametric Mann–Whitney test for independent groups.

We used a 256 mN stimulus and a 32 mN stimulus to induce mechanical stimulations to the lower back and hand of all participants. For chronic pain patients, the lower back region represents the site of their chronic pain. Pain scores across all trials per stimulus intensity across all groups were analyzed and compared using the Mann–Whitney U test; both participants with CLBP, as well as control participants, reported higher pain scores with the 256 mN stimulus than the 32 mN stimulus at both the hand (3.1 [2.3–4.6] vs. 1.3 [0.7–2.4] and 1.9 [1.0–3.3] vs. 0.7 [0.3–1.1], respectively: *p* < 0.001) and the lower back (3.3 [2.3–4.5] vs. 2.1 [1.2–2.8] and 2.0 [1.1–3.8] vs. 1.2 [0.4–2.1], respectively: *p* < 0.001). Importantly, as presented in Figure 1, participants with CLBP reported statistically significantly higher scores than control participants for the noxious stimuli at both the lower back and hand (*p* < 0.001).

### 3.3. Sensitivity to the site of chronic pain is associated with increased theta and alpha oscillations in the OFC

**Figure 2:**
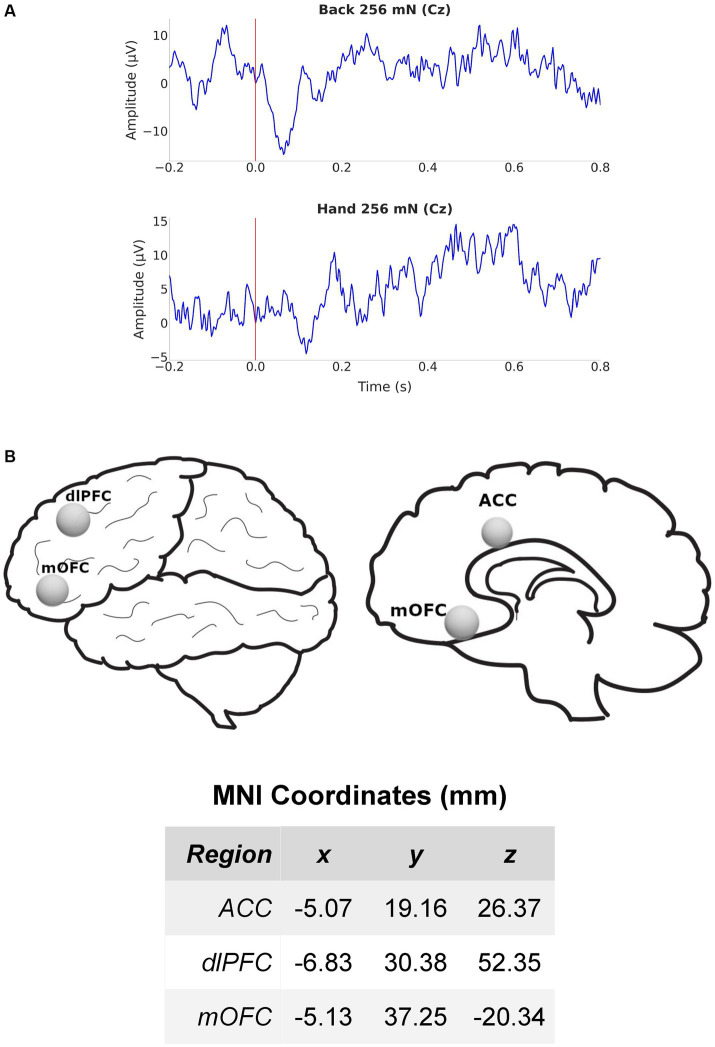
EEG recording and source localization overview. **(A)** Example EEG from electrode Cz in a chronic pain participant during mechanical stimulation (256 mN) to the lower back (top) and hand (bottom). **(B)** Lateral and medial views of the brain and regions of interest (ROIs) used for source localization. Also shown are the MNI coordinates of the 3 (contralateral) ROIs in millimeter scale. ACC, anterior cingulate cortex; dlPFC, dorsolateral prefrontal cortex; mOFC, medial orbitofrontal cortex.

We measured cortical activity using a 64-channel-EEG cap, for 5 min at baseline before and during mechanical stimulations to the lower back of all participants. EEG signals were sampled at 1000 Hz and band-pass filtered between 1 and 100 Hz. After band-pass filtering, we source localized our data using dSPM (Figure 2). To better understand mechanisms for both site-specific and anatomically nonspecific hyperalgesia, we focused our analysis on cortical areas such as the ACC, dlPFC, and mOFC which are known to be important for pain processing but demonstrate little or no somatotopy.

**Figure 3:**
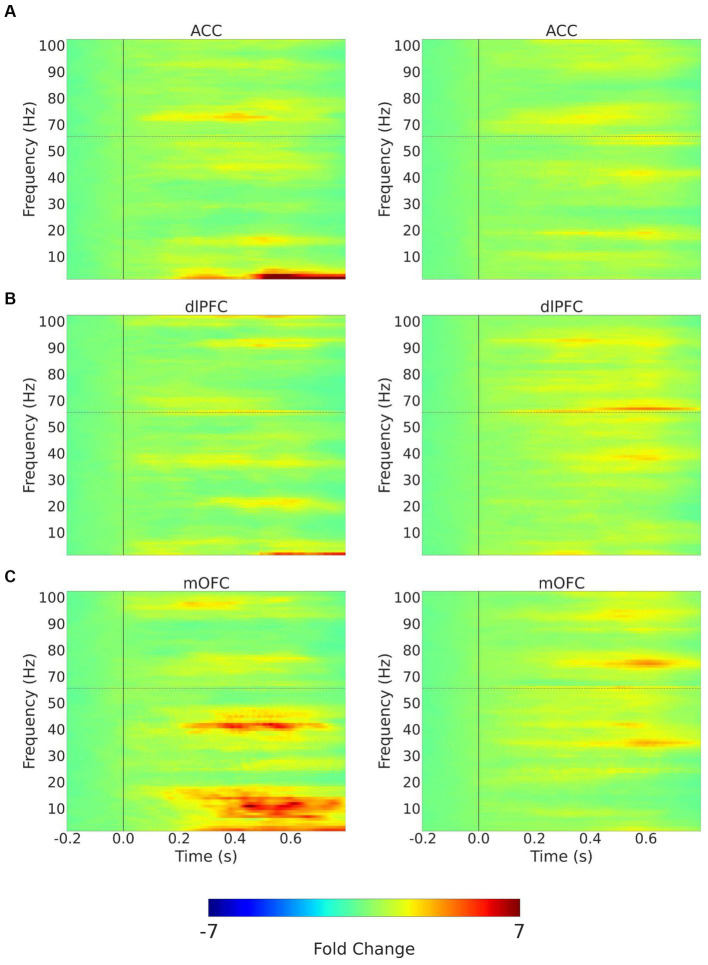
Trial-averaged time-frequency spectra with mechanical stimulation to the lower back with 256 mN. Time-frequency spectra (TFRs) are shown for example recordings from a chronic pain participant (left) and a pain-free control participant (right) for the 3 (contralateral) ROIs, **(A)** ACC; **(B)** dlPFC; **(C)** mOFC. Frequencies between 55 and 65 Hz (horizontal dashed line) omitted to remove electrical line noise. The TFRs represent percent change with respect to the baseline (−0.2–0.0 s before stimulus onset). ACC, anterior cingulate cortex; dlPFC, dorsolateral prefrontal cortex; mOFC, medial orbitofrontal cortex.

**Figure 4:**
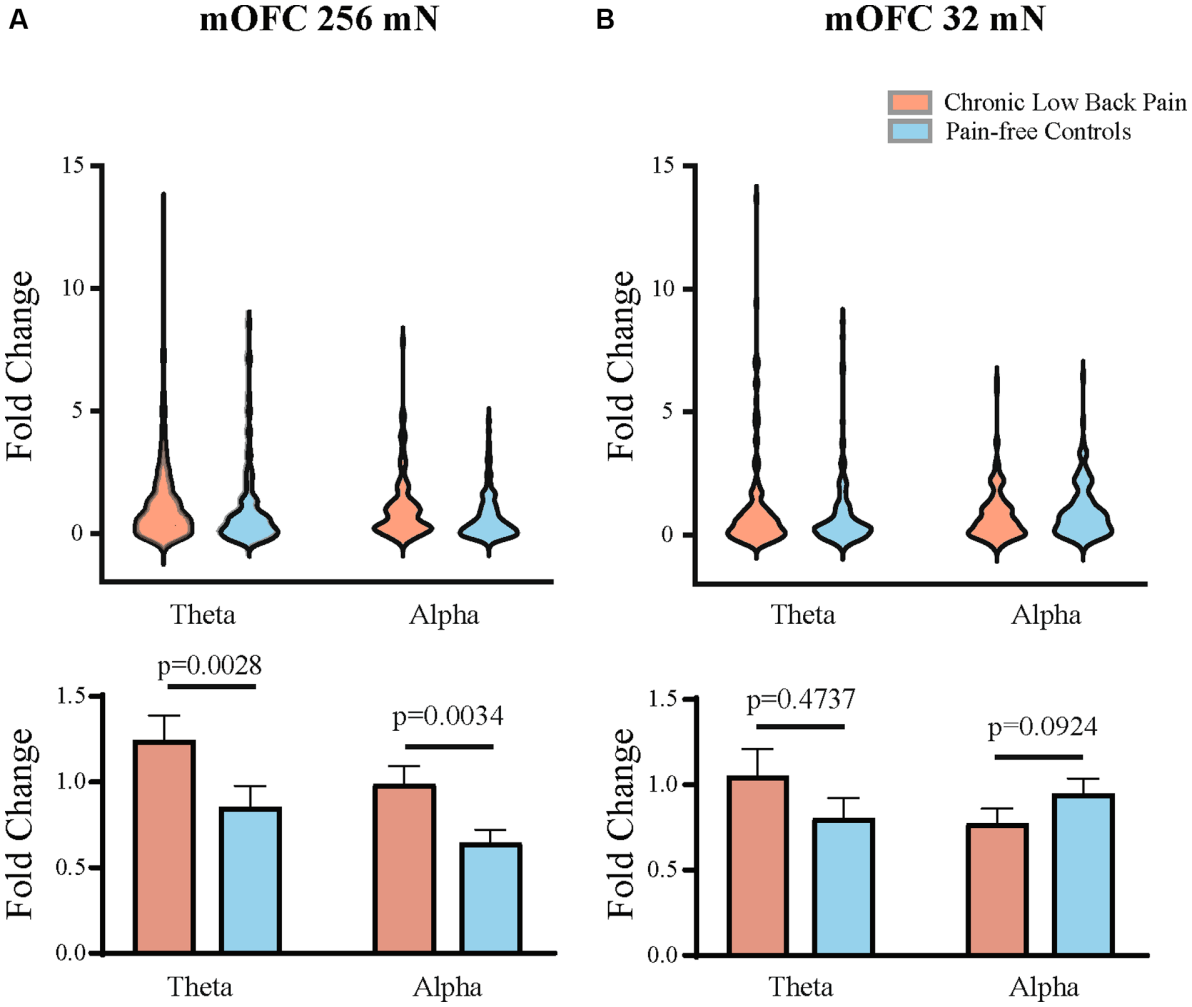
Mean theta and alpha band power are higher in the contralateral mOFC after mechanical stimulations to the lower back. **(A)** Mechanical stimulation with 256 mN results in a greater increase in the fold change of mean power in the theta (*p* = 0.0028) and alpha frequencies (*p* = 0.0034) of mOFC in participants with chronic low back pain (*n* = 15 participants and 144 trials) than pain-free controls (*n* = 15 participants and 143 trials). **(B)** Stimulation with 32 mN did not result in any statistically significant differences in the theta and alpha frequencies between chronic pain (*n* = 15 participants and 151 trials) and pain-free control participants (*n* = 15 participants and 143 trials). Data are shown as mean +/− SEM.

We quantitated EEG power in the control participants and CLBP participants, and measured power in canonical frequency bands while focusing on theta (4–8 Hz), alpha (8–13 Hz), and high-gamma (65–100 Hz) bands (Figure 3; Supplementary Figure S1). We then calculated changes in power from baseline in these frequencies in response to 32 mN or 256 mN stimuli applied to the back of these participants. We found that participants with CLBP demonstrated a statistically significant increase in the mean power at the alpha and theta frequencies in the contralateral medial OFC, compared with pain-free control participants (Figure 4) for the 256 mN stimulus (theta 1.243 ± 0.1453 vs. 0.8538 ± 0.1239, *p* = 0.0028, alpha 0.9887 ± 0.1040 vs. 0.6394 ± 0.0775, *p* = 0.0034) but not the 32 mN stimulus (theta 1.0494 ± 0.1606 vs. 0.8022 ± 0.1227, *p* = 0.4737, alpha 0.7735 ± 0.0857 vs. 0.9466 ± 0.0902, *p* = 0.0924).

### 3.4. Sensitivity to anatomically unrelated sites among chronic pain participants is associated with increased theta in dlPFC and gamma oscillations in the ACC

**Figure 5:**
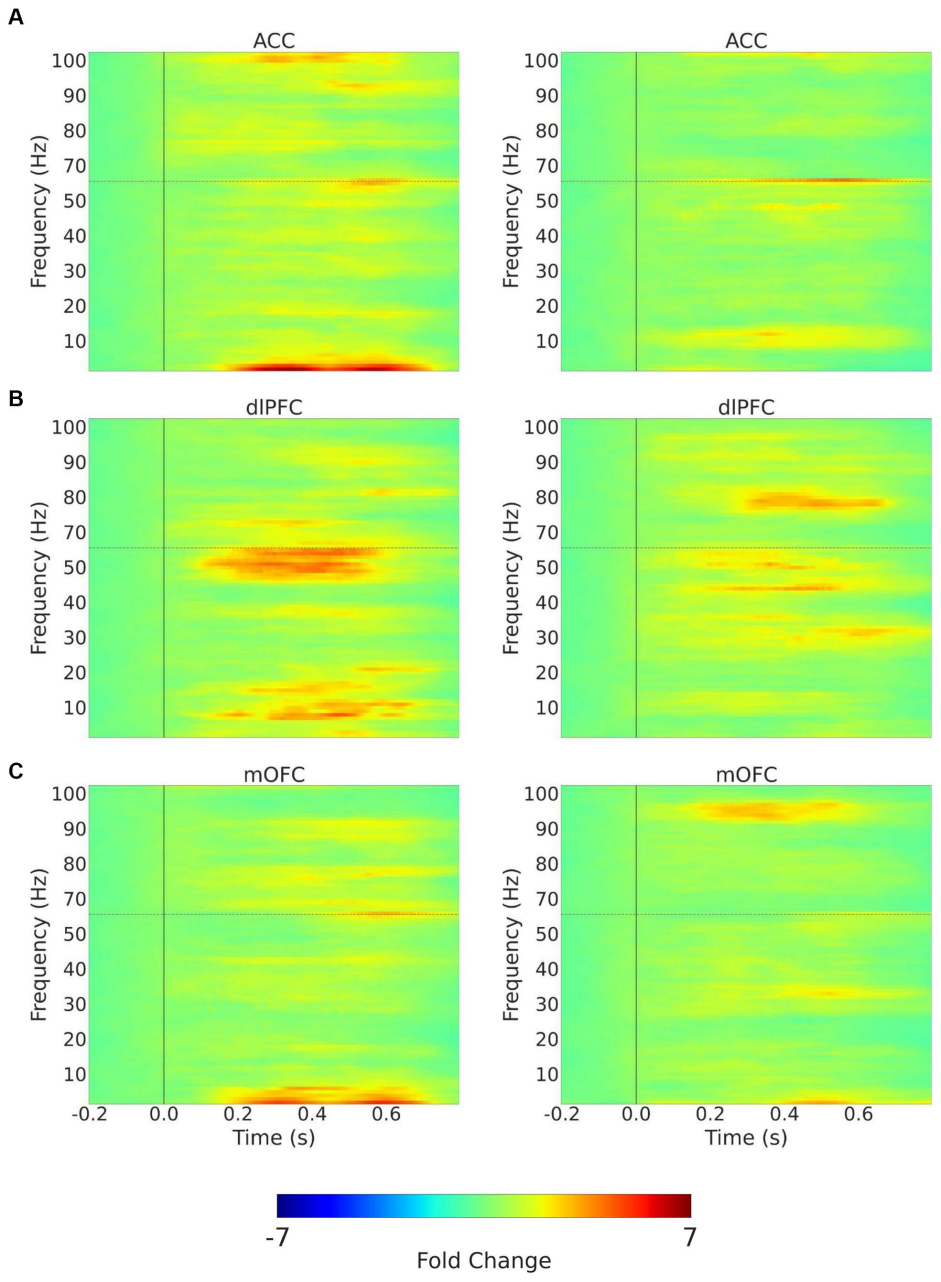
Trial-averaged time-frequency spectra with mechanical stimulation to the hand with 256 mN. Time-frequency spectra (TFRs) are shown for example recordings from a chronic pain participant (left) and a pain-free control participant (right) for the 3 (contralateral) ROIs, **(A)** ACC; **(B)** dlPFC; **(C)** mOFC. Frequencies between 55 and 65 Hz (hoirizontal dashed line) omitted to remove electrical line noise. The TFRs represent percent change with respect to the baseline (−0.2–0.0 s before stimulus onset). ACC, anterior cingulate cortex; dlPFC, dorsolateral prefrontal cortex; mOFC, medial orbitofrontal cortex.

**Figure 6:**
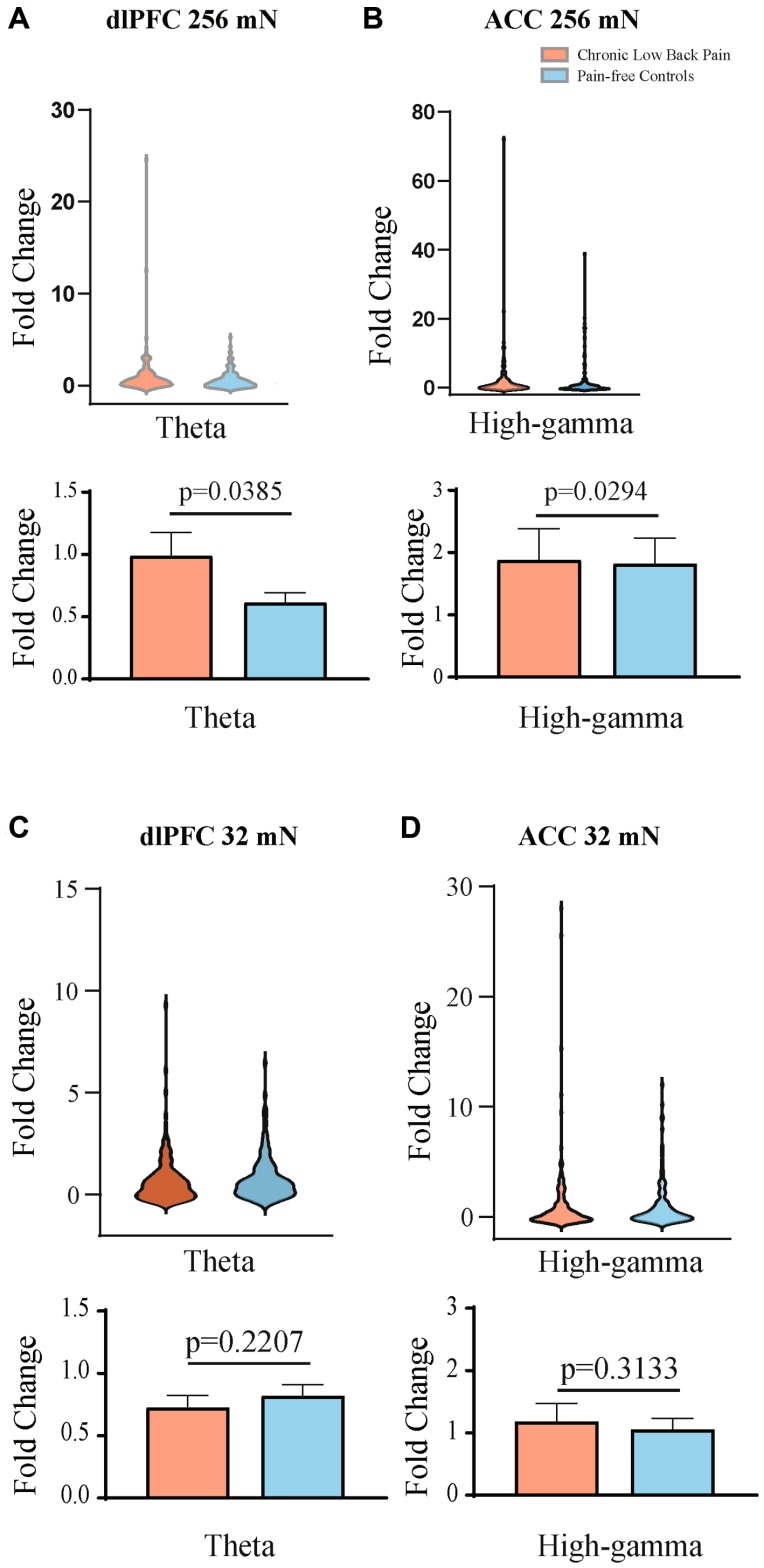
Mean theta and high gamma band power are higher in the contralateral dlPFC and contralateral ACC, respectively, after mechanical stimulations to the hand. **(A)** Mechanical stimulation with 256 mN results in a greater fold increase in the mean power of the theta frequency band in the dlPFC of participants with chronic low back pain (*n* = 15 participants and 156 trials) than that of pain-free controls (*n* = 15 participants and 148 trials), *p* = 0.0385. **(B)** Mechanical stimulation with 256 mN results in a greater fold increase in the mean power of the high-gamma frequency band in the ACC of participants with chronic low back pain than that of pain-free controls, *p* = 0.0294. **(C)** Stimulation with 32 mN did not result in any statistically significant differences in the mean power of the theta frequency band in the dlPFC between chronic pain and pain-free control participants. **(D)** Stimulation with 32 mN did not result in any statistically significant differences in the mean power of the high-gamma frequency band in the ACC between chronic pain (*n* = 15 participants and 156 trials) and pain-free control participants (*n* = 15 participants and 147 trials). Data are shown as mean +/− SEM.

Next, we quantitated EEG powers in the control participants and CLBP participants, and measured power in the theta (4–8 Hz), alpha (8–13 Hz), and high-gamma (65–100 Hz) bands in response to mechanical stimulations to the hand of all participants (Figure 5; Supplementary Figure S2). We calculated changes in power from baseline across different frequencies in response to either 32 mN or 256 mN stimuli applied to the right hand of these participants. We found that participants with CLBP, compared with control participants, demonstrated a statistically significant increase in the mean power at the theta frequency in the dlPFC and the high gamma frequency band in the ACC (Figure 6) with the 256 mN stimulus (theta 0.9909 ± 0.1872 vs. 0.6138 ± 0.0799, *p* = 0.0385, high-gamma 1.8779 ± 0.5073 vs. 1.8198 ± 0.4141, *p* = 0.0294) but not the 32 mN stimulus (theta 0.7257 ± 0.0979 vs. 0.8179 ± 0.0915, *p* = 0.2207, high-gamma 1.1838 ± 0.2862 vs. 1.0532 ± 0.1777, *p* = 0.3133).

## 4. Discussion

In this study, we used EEG to record cortical responses to acute mechanical stimuli applied to participants with CLBP vs. pain-free control participants. Our data indicate that CLBP participants demonstrated hypersensitivity both at the site of chronic pain and at a non-painful site. Source localized EEG data, meanwhile, suggest distinct cortical mechanisms that underlie hypersensitivity to painful and non-painful sites in these participants.

There is accumulating evidence that specific oscillatory activity patterns in anatomically defined brain regions, as well as the synchronization between them, play a key role in acute and chronic pain processing. The use of source localization algorithms for high-density EEG recordings, in particular, is an emerging technical development in the pain field that has enabled insights into cortical mechanisms of both acute and chronic pain in human subjects (Stern et al., 2006; Jensen et al., 2013; Prichep et al., 2018; Seeber et al., 2019; Teixeira M. et al., 2021; Teixeira P. E. P. et al., 2021; Völker et al., 2021; Mussigmann et al., 2022; Bott et al., 2023; Rockholt et al., 2023). In this study, we have focused on the cortical response to evoked stimuli rather than resting measurements. There have already been a number of EEG studies examining resting cortical activity in pain states, and changes in power responses in various frequency bands have been reported (Stern et al., 2006; Michels et al., 2011; Van Den Broeke et al., 2013; Prichep et al., 2018; Teixeira M. et al., 2021; Teixeira P. E. P. et al., 2021; Vanneste and De Ridder, 2021; Heitmann et al., 2022; Rockholt et al., 2023). However, the evaluation of resting state EEG potentials can sometimes be confounded by other time-invariant brain processes (Hansen et al., 2017). In contrast, evoked EEG provides time-sensitive data, and thus can complement findings from resting EEG studies; in particular, evoked EEG allows assessment of how the presence of chronic pain alters normal nociceptive processing (Teixeira M. et al., 2021; Teixeira P. E. P. et al., 2021) and thus provide insights into mechanisms underlying pathological processes such as allodynia and hyperalgesia (Plaghki and Mouraux, 2005). This information is of particular interest when examining chronic pain participants, as the number of studies evaluating neural responses to evoked painful stimuli in this cohort is scarce (Apkarian et al., 2005; Mussigmann et al., 2022).

In this study, we found changes in alpha, theta and gamma oscillations in chronic pain participants, and indeed oscillatory activities in these frequency ranges have been repeatedly shown to play important roles in previous studies on pain, particularly when evaluating evoked pain stimuli from pain-free participants (Gross et al., 2007; Zhang et al., 2012; Schulz et al., 2012b, 2015; Peng and Tang, 2016; Taesler and Rose, 2016; Peng et al., 2017). The alpha oscillation is the predominant oscillatory activity observed in sensory cortices at resting states and is commonly studied in the context of pain research. In resting state studies, increased alpha power has been noted in frontal, sensory, temporal, and parietal areas (Kisler et al., 2020). Experimentally induced transient pain in chronic pain and healthy cohorts elicited decreases in alpha power in the frontal-central or parietal-occipital regions which were inversely associated with stimulus intensity (Peng et al., 2014, 2015; Schulz et al., 2015; Misra et al., 2017; Nickel et al., 2017; Wang et al., 2019, 2021; Baroni et al., 2020). Theta oscillations in the hippocampus and cortex, however, are not only known to be important for sleep and cognition in animal and human studies (Green and Arduini, 1954; Winson, 1978; Wang, 2010; Seger et al., 2023), but have also been found to contribute to pain modulation (Taesler and Rose, 2016) where increased theta activity has been reported in participants with various chronic pain conditions, both in resting state recordings as well as evoked EEG potentials (Sarnthein and Jeanmonod, 2008; Vuckovic et al., 2014; González-Roldán et al., 2016; Hansen et al., 2017; Lee et al., 2017; Fallon et al., 2018; Wang et al., 2019). In addition, theta rhythmicity can also reflect a brain state of social response to social and fearful stimuli, further contributing to the pain experience (Tendler and Wagner, 2015). Meanwhile, changes in amplitudes of gamma oscillations have been shown to correlate with both evoked stimulus intensity and subjective pain rating in several studies (Gross et al., 2007; Michels et al., 2011; Zhang et al., 2012; Schulz et al., 2015; Mouraux and Iannetti, 2018; Zhou R. et al., 2018; Dinh et al., 2019; May et al., 2019; Baroni et al., 2020; Vanneste and De Ridder, 2021), where recent animal studies have also shown that theta and gamma oscillations in the ACC and S1 may encode the intensity of pain (Harris-Bozer and Peng, 2016; Tan et al., 2019; Sun et al., 2022, 2023; Zhang et al., 2023).

A key finding in our study is that whereas hypersensitivity at the site of injury (back) is associated with enhanced theta and alpha oscillations in the contralateral OFC, more generalized, anatomically-nonspecific enhancement in nociceptive response is seen with increased gamma oscillations in the ACC and increased theta oscillations in the contralateral dlPFC. To our knowledge, we are among the first group to distinguish between the locations of peripheral noxious stimuli. Such distinction is important, however, from both clinical and scientific viewpoints. Clinically, there is a need to distinguish between peripheral hypersensitivity and a more generalized, anatomically diffuse, enhancement for pain sensitivity, as treatments are often different. Scientifically, peripheral and spinal mechanisms as well as mechanisms in the brain may contribute to hypersensitivity at the site of injury, whereas the brain likely plays a more dominant role in a more generalized form of hyperalgesia. Our results here are compatible with findings from multiple animal studies that specifically investigated hyperalgesia of diffuse distribution, where enhancement in high gamma oscillatory activities in the ACC has been shown to be important and likely play a causal role (Zhang et al., 2017; Zhou H. et al., 2018; Friesner et al., 2020; Sun et al., 2021). The dlPFC, meanwhile, is known to produce top-down pain regulation, and its activation in response to nociceptive inputs has been widely reported in both human and animal literature (Hardy, 1985; Lee et al., 2015; Zhang et al., 2015; Kiritoshi et al., 2016; Martinez et al., 2017; Dale et al., 2018). Interestingly, a recent study using chronic intracranial recordings in patients with refractory pain showed that sustained power changes from the OFC can be used to detect the presence of chronic pain, whereas transient, evoked pain processing may be found in the ACC (Shirvalkar et al., 2023). It is possible that peripheral hypersensitivity serves as an index of chronic pain, and that OFC, which has prominent roles in reward processing as well as placebo analgesia (Petrovic et al., 2002; Kringelbach, 2005), may integrate multiple cognitive functions including disease-threat assessment to process allodynic-type of experiences. Due to the lack of threat at the non-injured site, OFC activity may play a more minor role, as shown by results in the cortical response to hand stimulation in our study.

There are several limitations to this study. The sample size was limited, and thus it is possible that the study was not powered to detect changes in all cortical areas. Further, the study was focused on participants with CLBP, and there are many additional chronic pain conditions. Future investigations with larger sample sizes and the inclusion of other chronic pain conditions are needed to assess the generalizability of our findings. Large sample sizes may also facilitate functional connectivity studies to understand how nociceptive information flows among different regions. Further studies of larger sample sizes are also needed to carefully analyze sex differences in cortical pain processing. Another limitation of this study is the use of two pinprick stimulators with fixed forces applied to all participants. Due to our sample size, we have based our EEG analysis on stimulus intensity, rather than pain scores, as done in previous studies (Nickel et al., 2017). Our protocol did not include temporal summation, and thus overall, evoked pain was less intense, unlike in previous studies of ERP responses to more intense stimulations (Hu et al., 2014). Hence, future studies of larger sample sizes using a larger range of noxious stimuli during EEG recordings could further analyze changes in brain activity. In addition, due to the nature of the short-lived mechanical stimulations, the cortical response we observed is likely the result of sensitization to A fiber stimulation, rather than C fiber stimulation, and thus future studies using different protocols such as thermal stimulations can provide a more complete picture of cortical changes in response to nociceptive inputs.

In conclusion, we have identified multiple cortical circuit elements that may underlie potential mechanisms on how chronic pain not only confers hypersensitivity at the site of injury but also induces a more anatomically nonspecific form of generalized hypersensitivity. Future studies of larger sample sizes utilizing more detailed analysis, including functional connectivity analysis, will provide further insights into how chronic pain alters normal nociceptive functions in the brain.

## Data availability statement

The original contributions presented in the study are included in the article/Supplementary material, further inquiries can be directed to the corresponding authors.

## Ethics statement

The studies involving humans were approved by Institutional Review Board of New York University Grossman School of Medicine. The studies were conducted in accordance with the local legislation and institutional requirements. The participants provided their written informed consent to participate in this study.

## Author contributions

GK: Data curation, Formal analysis, Methodology, Project administration, Software, Visualization, Writing – original draft, Writing – review & editing. MMR: Data curation, Formal analysis, Investigation, Project administration, Writing – original draft, Writing – review & editing. DO: Investigation, Project administration, Writing – review & editing. MM: Investigation, Project administration, Writing – review & editing. QZ: Data curation, Formal analysis, Methodology, Writing – review & editing. GS: Investigation, Methodology, Project administration, Writing – review & editing. JM: Data curation, Formal analysis, Writing – review & editing. AW: Data curation, Formal analysis, Writing – review & editing. BC: Data curation, Formal analysis, Writing – review & editing. EPV: Investigation, Project administration, Writing – review & editing. ZSC: Data curation, Formal analysis, Methodology, Writing – review & editing. JW: Conceptualization, Data curation, Formal analysis, Funding acquisition, Investigation, Methodology, Supervision, Writing – original draft, Writing – review & editing. LVD: Conceptualization, Data curation, Formal analysis, Funding acquisition, Investigation, Supervision, Writing – review & editing.
