# Supplementary figures and images for: Changes in alpha, theta, and gamma oscillations in distinct cortical areas are associated with altered acute pain responses in chronic low back pain patients
Source: Front Neurosci. 2023 Oct 13;17:1278183. doi: 10.3389/fnins.2023.1278183 (PMC10611481; doi:10.3389/fnins.2023.1278183)

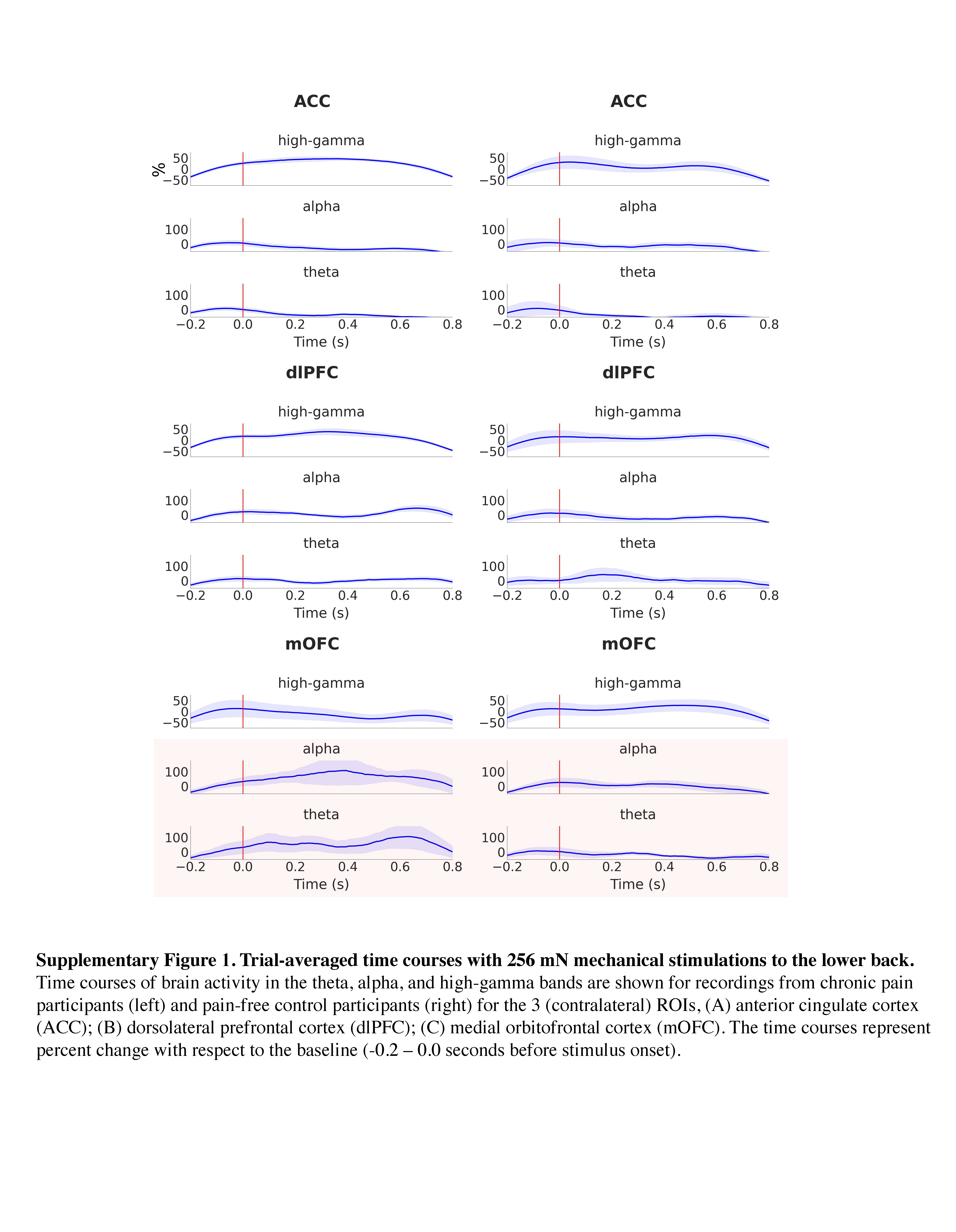

Supplement: Supplementary file 1 [file Image_1.TIFF]

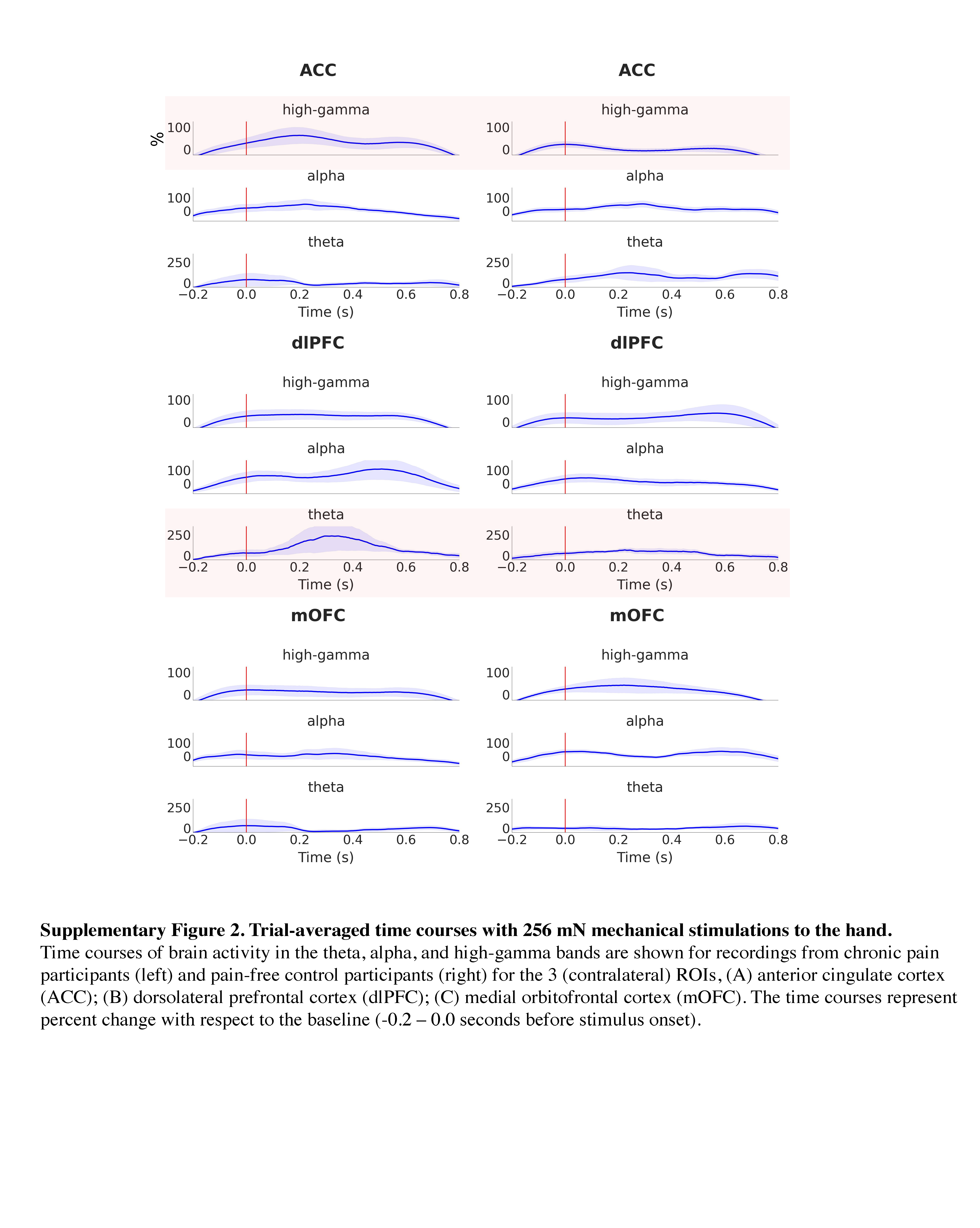

Supplement: Supplementary file 2 [file Image_2.TIFF]
